# Identification of Potential Functional Modules and Diagnostic Genes for Crohn’s Disease Based on Weighted Gene Co-expression Network Analysis and LASSO Algorithm

**DOI:** 10.5152/tjg.2025.23605

**Published:** 2025-01-06

**Authors:** Ruiquan Wang, Hongqi Zhao

**Affiliations:** 1 Department of Pathology, Jinhua People’s Hospital, Zhejiang, China; 2 Department of Pathology, Jinhua Guangfu Hospital, Zhejiang, China

**Keywords:** Crohn’s disease, inflammatory bowel disease, weighted gene co-expression network analysis, CIBERSORT, diagnostic model

## Abstract

**Background/Aims:**

Accurate diagnosis of Crohn’s disease (CD) is paramount due to its resemblance to other inflammatory bowel diseases. Early and precise diagnosis plays a pivotal role in tailoring personalized treatments, thereby enhancing the quality of life for CD patients.

**Materials and Methods:**

Differential gene expression analysis was conducted to identify genes from the mRNA expression profiles of CD samples, followed by pathway enrichment analysis. The immune cell infiltration levels of each CD patient sample were assessed. Using weighted gene co-expression network analysis, key gene modules linked to CD were found. Hub gene identification was made easier by the construction of protein–protein interaction networks. Next, utilizing the Least Absolute Shrinkage and Selection Operator on the hub genes in the training set, a diagnostic model was created. The accuracy of the model was then confirmed using a different validation set.

**Results:**

Our analysis revealed 651 differentially expressed genes, enriched in leukocyte chemotaxis and inflammation-related pathways. Immunization results showed a higher abundance of T cells CD4 memory resting, macrophages M2, and plasma cells in CD patients. Weighted gene co-expression network analysis linked the turquoise module with macrophages M2. Eight hub genes (APOA1, APOA4, CYP2C8, CYP2C9, CYP2J2, EPHX2, HSD3B1, and LPL) formed the diagnostic model, exhibiting excellent diagnostic performance with area under curve values of 0.94 (training set) and 0.941 (validation set).

**Conclusion:**

The CD diagnostic model, based on hub genes, shows exceptional diagnostic accuracy, providing a valuable reference for CD diagnosis.

**Main Points**

- The study established an 8-gene diagnostic model for Crohn’s disease, showing superior capability in Crohn’s disease diagnosis.
- Genes in patients with Crohn’s disease were found to be mainly enriched in inflammation-related pathways.
- The abundance of macrophages M2 was higher in patients with Crohn’s disease.

## Introduction

Crohn’s disease (CD) is one of the inflammatory bowel diseases (IBD). Unlike other inflammatory diseases, CD is characterized by frequent relapses that are difficult to control, predominantly affecting patients between the ages of 15 and 35.^1^ Stimulation of the immune system induces partial destruction of the intestinal mucosa, resulting in symptoms such as diarrhea, rectal bleeding, weight loss, fever, abdominal pain, and abdominal cramps, leading to a decreased quality of life.^2, 3^

The diagnosis of CD is primarily based on clinical manifestations, histology, endoscopy, radiology, and/or biochemical examinations.^4^ Although a complete pathological examination can provide a more accurate diagnosis, it is challenging to perform biopsies on patients during disease flares. Furthermore, distinguishing CD from other intestinal diseases based on endoscopic examinations, CT scans, and histological features is difficult in clinical practice.^5,6^ Delayed diagnosis and treatment of CD due to difficulties in differentiation can lead to severe complications such as intestinal perforation and fistula.^7^ Early diagnosis is crucial to ensure timely medical intervention, control disease progression, and improve patients’ quality of life. Therefore, making the correct differential diagnosis for CD is of paramount importance.

Weighted gene co-expression network analysis (WGCNA) is applied to mine module information from gene expression data obtained from microarrays.^8^ It allows for gene clustering, forming modules based on similar gene expression patterns to explore the association between modules and specific features, such as patients’ clinical information.^8^ Currently, WGCNA has proven to be effective in identifying therapeutic targets and biomarkers in various diseases. Ai et al^9^ constructed a diagnostic model based on 10 genes using asthma-related features, which demonstrates good diagnostic performance. Hsueh et al^10^ developed an optimal diagnostic model for a hereditary neuromuscular disorder through the screening of clinical patients, achieving higher diagnostic rates compared to other studies, with detection rates exceeding 70% in certain cases. It is evident that WGCNA is a powerful tool that enables researchers to uncover hidden patterns and correlations in gene expression data, thereby elucidating gene regulatory mechanisms, identifying biomarkers, predicting gene functions, and providing novel insights and revelations for biological research and disease diagnosis. In this study, we aim to apply WGCNA to extract key genes linked to CD and construct a diagnostic model to assist clinicians in making accurate diagnoses of CD.

In this investigation, we analyzed gene expression data related to CD from Gene Expression Omnibus (GEO). Immunocytes related to CD were selected as epigenetic data. Utilizing WGCNA and Least Absolute Selection and Shrinkage Operator (LASSO) logistic regression, we aimed to screen out key genes and build a diagnostic model to provide clinical reference for CD diagnosis (Figure S1).

## Materials and Methods

### Data Download and Differentially Expressed Genes Identification

The GSE186582 dataset (annotation platform: GPL570) containing 196 CD samples and 25 control samples was downloaded from GEO (https://www.ncbi.nlm.nih.gov/). Differential analysis was done utilizing “limma” package (Version 3.54.2) with a threshold set at |log FC| > 1 and adjust_*P* < .05 to identify differentially expressed genes (DEGs). For microarray data, this study utilized the normalizeBetweenArrays function for normalization. Following normalization, linear model fitting and gene detection were performed using the lmFit and eBayes functions.

### Kyoto Encyclopedia of Genes and Genomes and Gene Ontology Enrichment Analyses

Gene ontology (GO) enrichment analysis is a method of annotating gene products, providing insights into the biological processes, molecular functions, and cellular locations in which genes are enriched. Kyoto Encyclopedia of Genes and Genomes (KEGG) enrichment analysis is a systematic analysis of gene products involved in cellular metabolic pathways. We utilized the “clusterProfiler” package (Version 4.6.2) to do GO and KEGG enrichment analyses on DEGs in CD (*P* < .05), identifying the most significantly enriched biological functions and signaling pathways.

### CIBERSORT

Based on the concepts of linear support vector regression, CIBERSORT is a deconvolution technique applied to human immune cell subtype expression matrices. According to transcriptome data, CIBERSORT can estimate the composition and abundance of human immune cells. By analyzing the relative expression levels of genes in individual tissue samples based on gene expression profiles, CIBERSORT determines expression of 22 tumor-infiltrating immune cells in each sample. During this process, a multiple testing correction was performed 1000 times. Monte Carlo sampling is used to derive a *P*-value as a measure of confidence for each sample’s deconvolution. Samples with immune cell inference from CIBERSORT that have a *P* < .05 are statistically significant. Subsequently, statistical results from CIBERSORT are used to filter the epigenetic data for WGCNA analysis. Ethics Committee Approval and Informed Consent were not required as this study was based on publicly available data.

### Weighted Gene Co-expression Network Analysis

“WGCNA” package (Version 1.72-1) was employed for further analysis of DEGs to build a scale-free co-expression network. A soft threshold of *β* = 9 was chosen to ensure a scale-free network. Each module’s gene information was retrieved, with a minimum gene count of 10. A hierarchical clustering dendrogram was produced using the blockwiseModules function. Branches of the clustering dendrogram were combined into distinct gene modules using the dynamic tree cut approach. Various colors were used to depict the resultant modules. Additionally, “pheatmap” package (Version 1.72-1) was employed to visualize correlations between epigenetic data and gene modules and to identify significant gene modules.

### The Establishment of a Protein–Protein Interaction Network and Selection of Hub Genes

Genes were extracted from significant modules. The Search Tool for the Retrieval of Interacting Genes (STRING; http://string.embl.de/) was employed to construct a protein–protein interaction (PPI) network. The minimum confidence score was set at 0.9 to obtain important genes. The hub genes were selected by utilizing 5 algorithms (Maximum Neighborhood Component (MNC), Maximal Clique Centrality (MCC), Density of Maximum Neighborhood Component (DMNC), Edge Percolated Component (EPC), and Degree) in the cytoHubba plugin of Cytoscape software.

### The Screening and Validation of Diagnostic Biomarkers

The create Data Partition function from the caret package (Version 6.0-94) was used to divide the samples into training and validation sets at random in a 7:3 ratio. A LASSO logistic regression diagnostic model was created with hub genes for the training data. Model diagnostic ability was determined by plotting receiver operating characteristics (ROC) curve and computing area under the curve (AUC) value. Model was further validated using the validation set.

## Results

### Differentially Expressed Genes and Their Functional Enrichment in Crohn’s Disease

**Figure 1.**
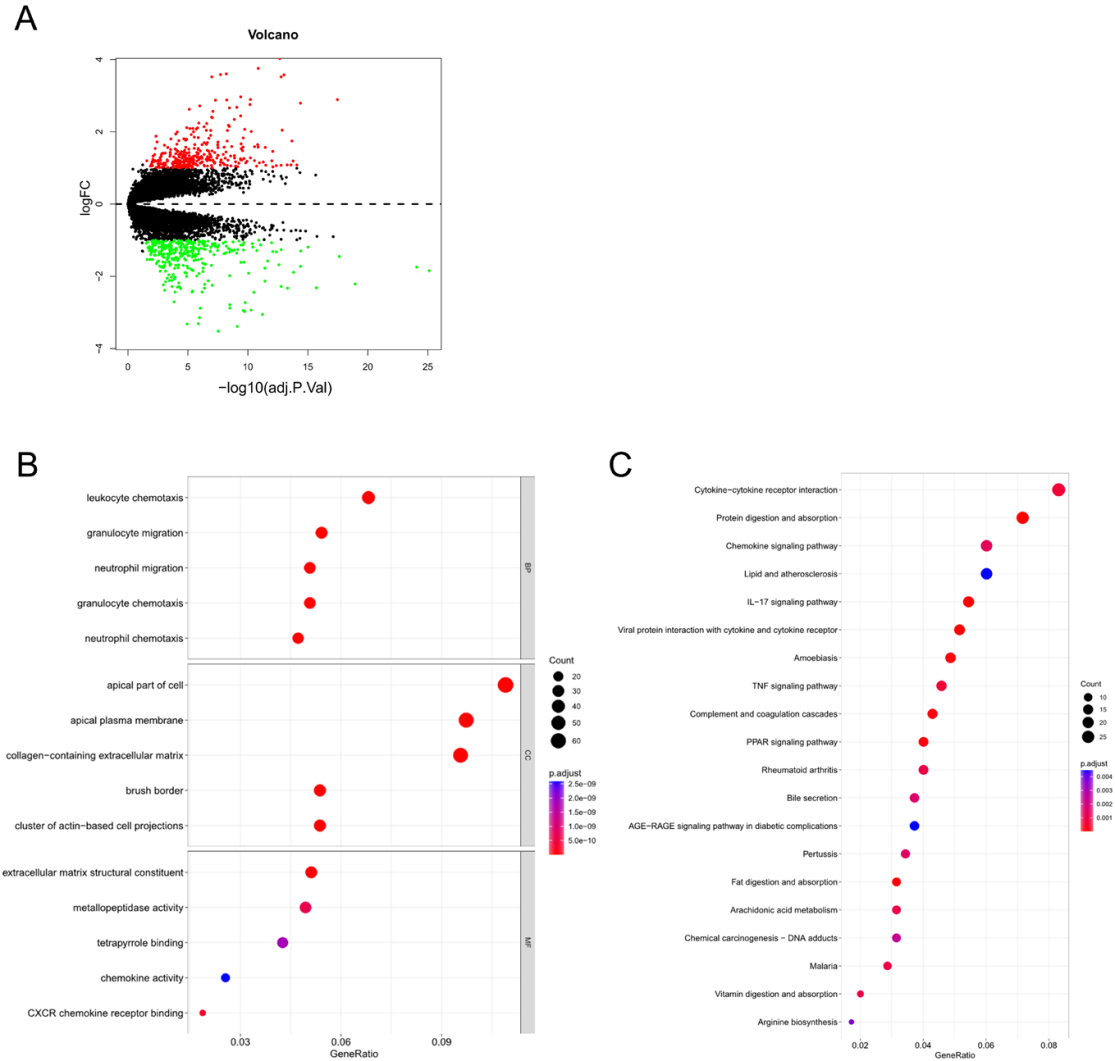
DEGs and functional enrichment analyses in CD samples. (A) Volcano plot of DEGs between CD and control samples. Red dots indicate significantly up-regulated genes and green dots indicate significantly down-regulated genes. Black dots indicate non-significantly DEGs. (B, C) Results of GO (B) and KEGG (C) enrichment analyses. The abscissa represents the proportion of genes in the pathway. The ordinate indicates the pathway enriched by the gene. Dot size indicates the number of genes enriched in the pathway.

In order to understand the functions of DEGs related to CD and elucidate the pathogenesis of CD, we conducted enrichment analysis. Differential analysis was performed on 196 CD samples and 25 control samples from the GEO dataset, resulting in 651 DEGs (Supplementary Table 1) (Figure 1A). To further figure out the potential functions and pathways related to CD patients, GO and KEGG were carried out on these DEGs. These genes were predominantly enriched in biological functions such as leukocyte chemotaxis, apical part of cell, and extracellular matrix structural constituent, according to GO results (Figure 1B). They were most abundant in pathways including cytokine–cytokine receptor interaction, chemokine signaling pathway, IL-17 signaling pathway, TNF signaling pathway, and viral protein interaction with cytokine and cytokine receptor, as revealed by KEGG results (Figure 1C). The DEGs in CD are primarily involved in inflammation-related pathways.

### The Calculation of Immune Cell Composition and Selection of Epigenetic Data in Crohn’s Disease Samples

**Figure 2.**
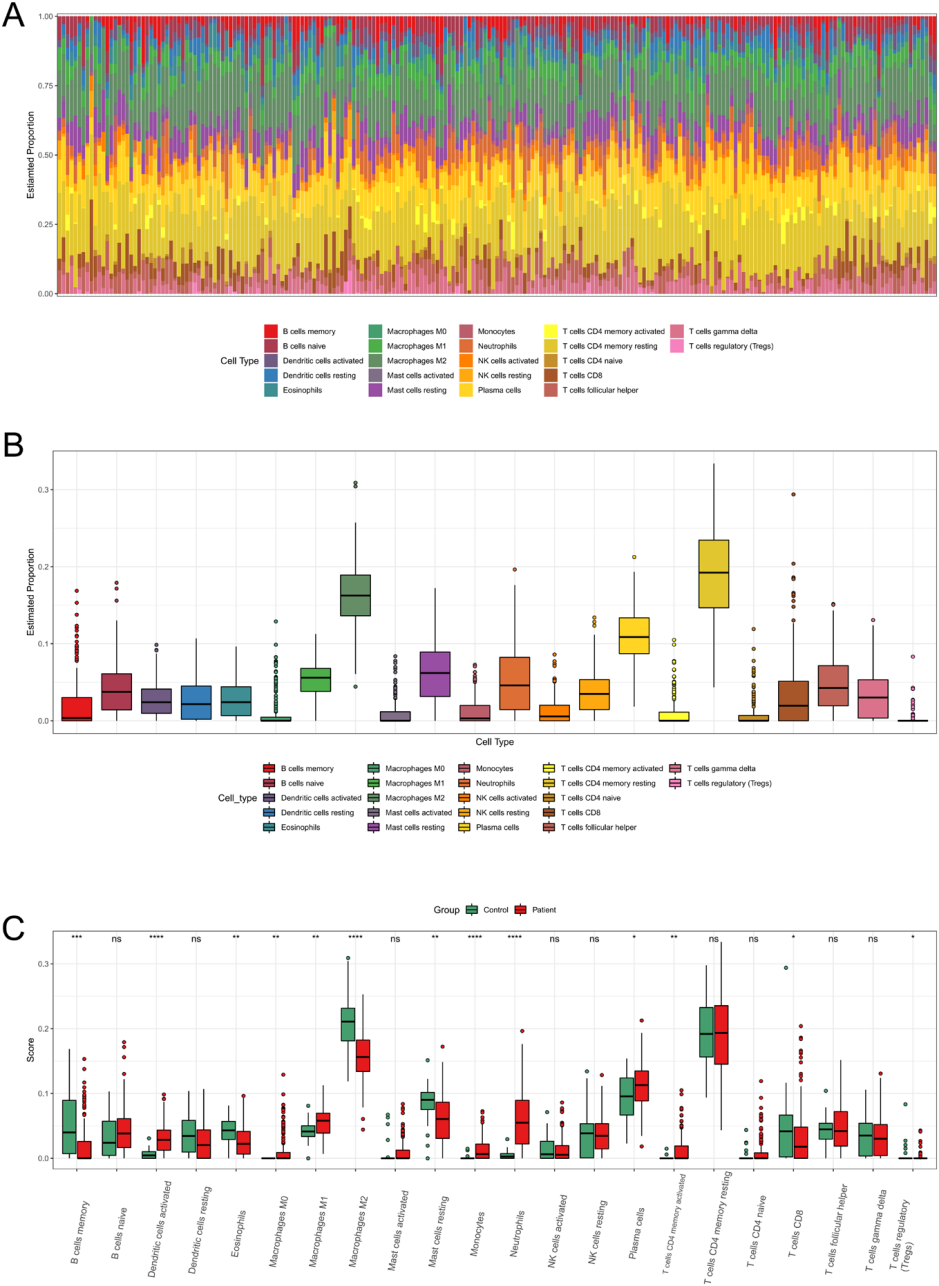
Results of CIBERSORT analysis. (A) Immune cell abundance in each CD sample. The abscissa represents the number of patients, and the ordinate represents the proportion of infiltration of different cell types in patients. (B) Differential analysis of immune cell proportions in CD. The abscissa represents the cells, and the ordinate represents the infiltrating proportion of the cells. (C) Differential analysis of immune cells between the control and CD groups. Cells are represented on the abscissa and cell infiltration scores are represented on the ordinate.

The aberrant immune system activation is intimately linked to the pathophysiology of CD. Researching the connection between CD and immunity can provide us a theoretical framework for developing treatment approaches that target immune regulation by illuminating the immune system’s function in the initiation and progression of the illness. To this end, CIBERSORT algorithm was employed to test immune cell composition in CD samples. A heatmap was utilized to visualize the results (Figure 2A). As revealed by the proportion statistics, the proportions of macrophages M2, plasma cells, and T cells CD4 memory resting were higher in CD patients (Figure 2B). Differences in each immune cell type between the control and CD groups were analyzed. The results revealed that B cells memory, eosinophils, macrophages M2, mast cells resting, and T cells CD8 were considerably increased in the control group compared to CD patients. Macrophages M1, monocytes, plasma cells, neutrophils, and T cells CD4 memory activated were considerably lower in control group compared to CD patients (Figure 2C). Previous studies have pointed out that an increased abundance of macrophages M2 is linked to the resolution or improvement of chronic inflammation, being capable of alleviating the symptoms of IBD.^11^ Therefore, we selected macrophages M2 as the epigenetic data for further analysis in CD.

### The Selection of Crohn’s Disease-Related Key Modules Using Weighted Gene Co-expression Network Analysis

**Figure 3.**
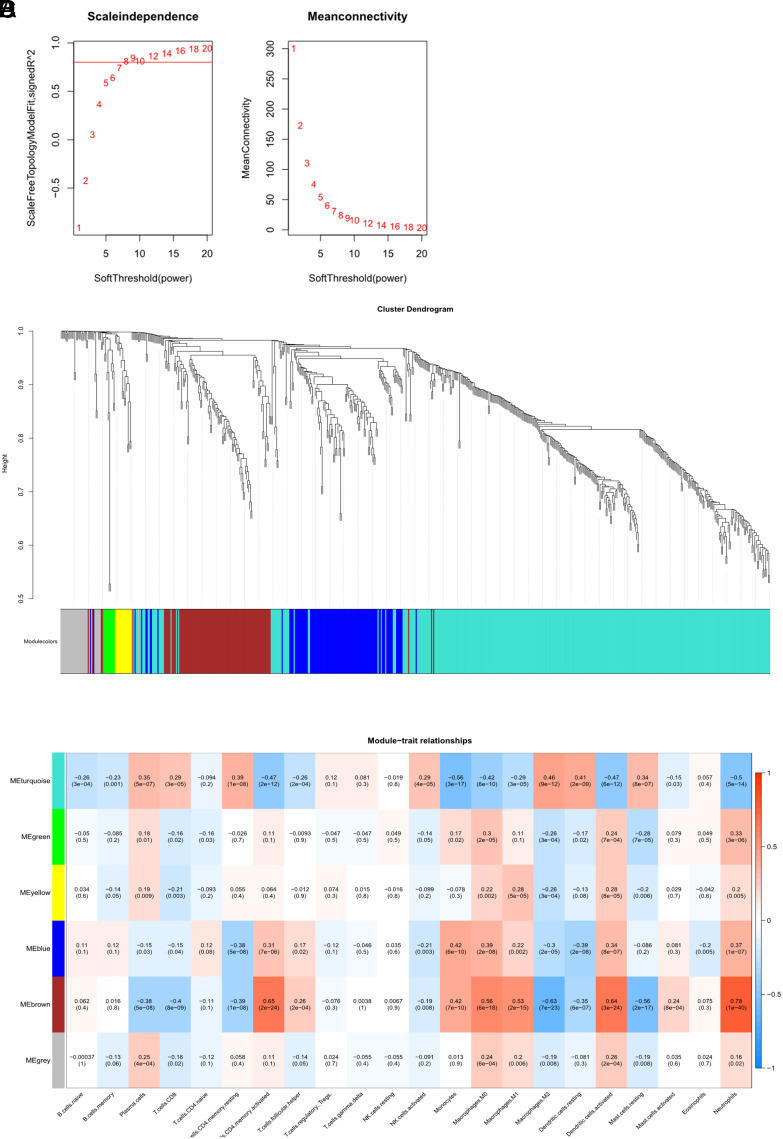
The construction of the WGCNA and identification of key modules. Scale-free fitting parameter for soft thresholding power. The abscissa represents the soft threshold, and the ordinate represents the scale-free topological fit index. (B) Network connectivity under different soft thresholding powers. The abscissa represents the soft threshold and the ordinate represents the connectivity. (C) Identification of modules by WGCNA. The bottom represents the module color to which the corresponding tree branch is assigned, and each color represents a module. (D) Heatmap showing the correlation between gene modules and immune cells. Red indicates positive correlation and blue indicates negative correlation.

To explore genes associated with CD, we conducted WGCNA analysis to find gene modules connected to the occurrence and course of CD. Further analysis was conducted on the aforementioned DEGs to construct a scale-free co-expression network (scale-free *R* > 0.8) with a soft thresholding power of 9 (Figure 3A and B). A clustering dendrogram was generated. Using the dynamic tree cut approach, the dendrogram’s branches were combined into 6 unique gene modules, each denoted by a different color (Figure 3C). The correlation coefficients were calculated to detect the correlation between each WGCNA module and immune cells. The ME turquoise module, which exhibited a relatively high correlation (0.46) with M2 macrophages, was selected as the focus module for subsequent analysis (Figure 3D). This module comprised 381 genes (Supplementary Table 2) that were used for further analysis.

### The Construction of a Protein–Protein Interaction Network and Selection of Hub Genes

**Figure 4.**
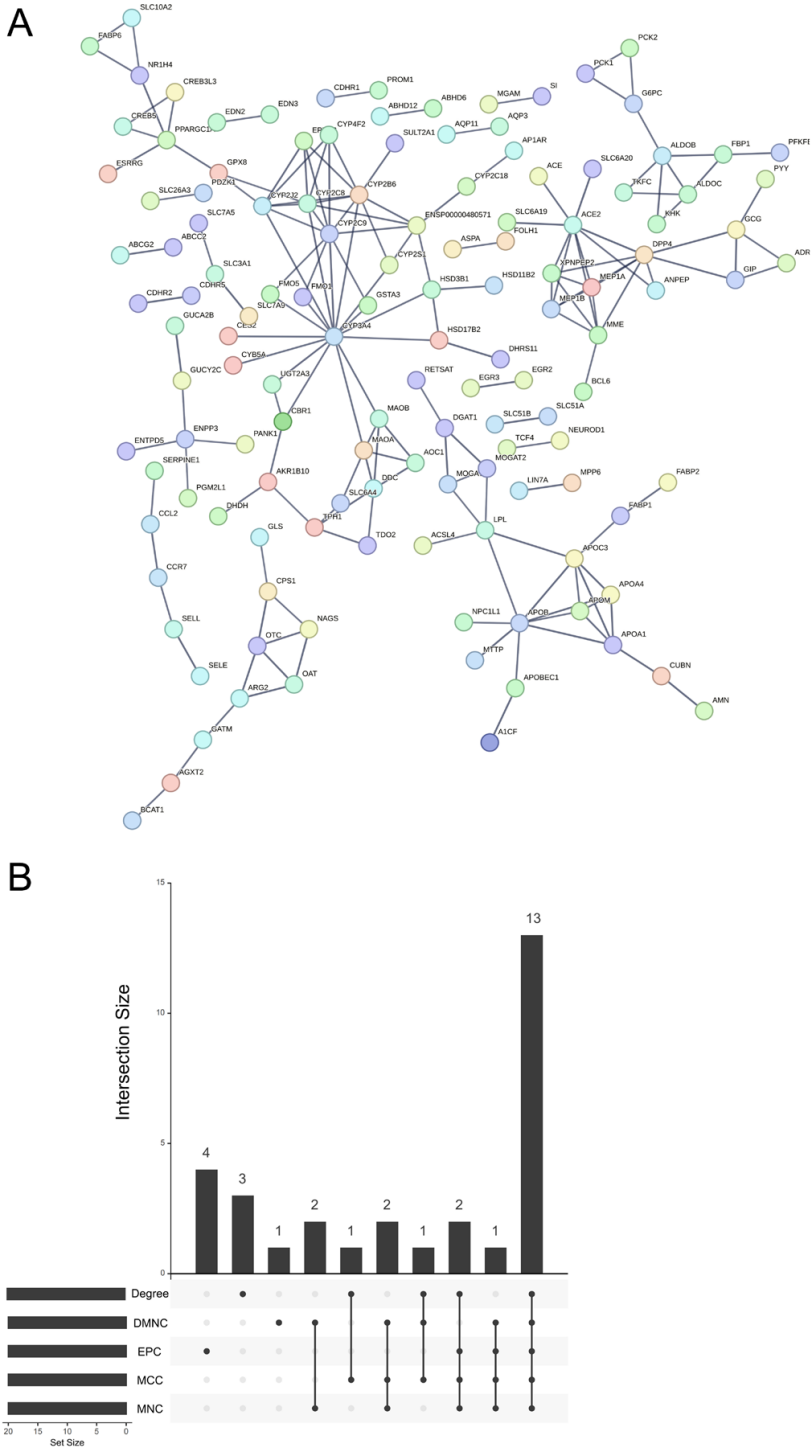
The selection of Hub genes. (A) A PPI network diagram. A node represents a protein, and a line between proteins indicates that the 2 proteins have a related role. (B) UpSet plot showing the intersection of hub genes selected by the cytoHubba plugin. The lines between the dots represent the intersection genes of 2 or more algorithms.

To further screen out the core genes in the modules, we performed PPI analysis. The 381 genes from the ME turquoise module were utilized to establish a PPI network with a confidence score higher than 0.9 (Figure 4A). Network was subsequently subjected to hub gene selection by utilizing cytoHubba plugin in Cytoscape. The hub genes were found by considering the intersection of the genes that were chosen using the following 5 criteria: MNC, MCC, DMNC, EPC, and Degree. A total of 13 hub genes (APOA1, APOA4, APOB, APOC3, CYP2B6, CYP2C8, CYP2C9, CYP2J2, EPHX2, FABP1, HSD3B1, LPL, and PPARGC1A) were obtained (Figure 4B).

### The Construction and Validation of Diagnostic Model

**Figure 5.**
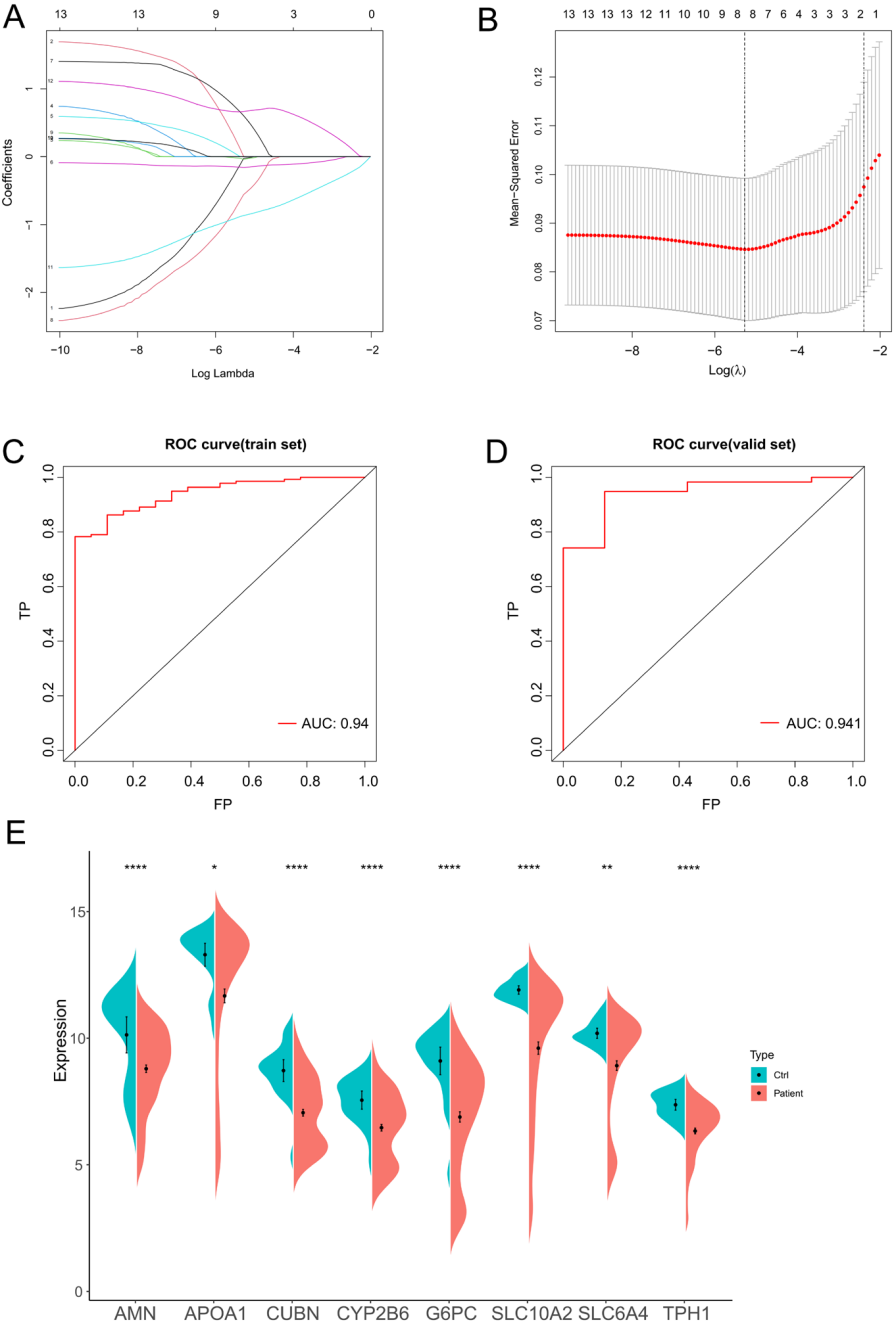
The selection of diagnostic biomarkers and model validation. (A) Coefficient distribution plot for logarithm (*λ*) sequence in the LASSO model. The abscissa represents the logarithmic *λ* value. The ordinate represents the coefficient of the gene. (B) LASSO coefficient spectrum. Two lambda values are obtained in the figure, and the lambda.min on the left refers to the value of *λ* at the minimum error. The lambd.1se on the right-hand side is the value of *λ* that yields the simplest model within a variance of lambd.min. The vertical dashed line in the right figure corresponds to the penalty value of the lowest point (that is, the upper coordinate corresponding to the lowest point of the curve), and the vertical line at the position of the corresponding penalty value in the left figure is searched for the corresponding position. The number of intersecting points is the number of variables included in the final model, and the vertical coordinate of the corresponding intersection point is the regression coefficient of the variable. (C, D) ROC curve analysis in the training set (C) and the validation set (D). The abscissa is the False Positive Rate, and the ordinate is the True Positive Rate. The area under the ROC curve can quantify the overall performance of the model, and the closer the AUC value is to 1, the better the overall performance of the model. (E) Differential analysis of hub genes in CD and control samples.

The 13 hub genes obtained by screening may be critical in CD progression. To explore diagnostic efficacy of these genes for CD, we performed the following analysis. According to a 7:3 ratio, dataset was grouped into training and validation sets. After LASSO regression analysis on the training set’s 13 hub genes, 8 hub genes—APOA1, APOA4, CYP2C8, CYP2C9, CYP2J2, EPHX2, HSD3B1, and LPL—were identified for use in building the diagnostic model (Figure 5A and B). The model formula is as follows. Coefficients for model genes were generated by LASSO regression analysis (Supplementary Table 3).



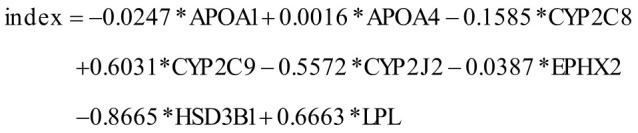



Receiver operating characteristics curve analysis and calculation of the AUC were conducted in the training set (Figure 5C), demonstrating a diagnostic efficiency of 0.94. Model diagnostic ability was validated in validation set, yielding a diagnostic efficiency of 0.941 (Figure 5D). These results indicated that the diagnostic model containing immune-related key genes exhibits good diagnostic capability for CD patients. Furthermore, the 8 hub gene levels were analyzed in both the control group and CD samples, revealing considerably elevated expression in the control group compared to CD samples (Figure 5E), further emphasizing the significant value of the selected hub genes for CD diagnosis.

## Discussion

Ngollo et al^12^ used dataset GSE186582 to identify genes associated with increased risk of postoperative recurrence in CD. In this study, the gene expression of patients in this dataset was used to identify the DEGs related to CD, and WGCNA and other methods were applied to screen 8 hub genes for constructing a CD diagnostic model, aiming to provide a new idea for the clinical diagnosis of CD.

We herein identified 651 DEGs associated with CD from the GEO dataset. The DEGs in CD are primarily involved in biological functions such as leukocyte chemotaxis. Chemokines and cytokines take crucial parts in regulating mucosal inflammation by facilitating migration of leukocytes to sites of inflammation, eventually resulting in tissue damage and destruction.^13^ Furthermore, CD is mainly involved in inflammatory response pathways such as TNF signaling and IL-17 signaling. Since inflammation is greatly implicated in the pathogenesis of CD, the inhibition of it can alleviate symptoms and prevent further tissue damage.^14^ Research by Louis et al^15^ has shown a significant increase in TNF-α in CD patients. Additionally, inhibition of TNF-α can effectively alleviate intestinal inflammation.^16^ Anti-TNF agents are among the first biologics approved for treating IBD and have been widely used for remission and treatment of CD patients.^17^ Anti-TNF treatment appeared to significantly ameliorate the pro-inflammatory phenotypes of macrophages and adipose-derived stem cells (ADSCs) in CD-switched ADSCs.^18^ IL-17 is a cytokine with strong pro-inflammatory activity. Studies have indicated that IL-17 mainly accumulates in the submucosal and muscularis propria layers of CD patients, possibly being associated with changes in intestinal mucosal immunity and inflammatory responses.^19,20^ Ashton et al^21^ showed that IL17 signaling was enriched in monocytes and epithelial cells. We found that the occurrence of CD is primarily associated with inflammation. IL-17 signaling and TNF signaling may serve as effective targets for CD treatment.

We identified 8 hub genes (APOA1, APOA4, CYP2C8, CYP2C9, CYP2J2, EPHX2, HSD3B1, and LPL) using methods such as WGCNA, PPI network, and LASSO logistic regression for constructing a diagnostic model. APOA1 and APOA4 are apolipoproteins of the apolipoprotein A family that can promote lipid transport and metabolism,^22^ exerting anti-inflammatory effects. CYP2C8 and CYP2C9 are drug-metabolizing enzymes involved in inactivation of various drugs,^23^ serving as functional enzymes to play a role in the independent regulation of intestinal function.^24^ CYP2J2 is majorly expressed in the intestine and can regulate intestinal motility and/or intestinal fluid/electrolyte transport.^25^ Research has found that the absence of CYP2J2 in CD macrophages may contribute to the development of CD.^26^ Reisdorf et al^27^ revealed that repression of EPHX2 significantly reduces IL-1β, a pro-inflammatory factor, in CD. HSD3B1 is a key member of the steroid hormone family.^28^ He et al^29^ constructed a CD prognosis model based on 4 genes, including HSD3B1, achieving good prognostic performance. Bo et al^30^ demonstrated that LPL is significantly upregulated in CD and may take a pivotal part in the pathogenesis of CD. Based on the above, the hub genes used in this study for constructing the diagnostic model are essential for the pathophysiology and development of CD, contributing to the clinical diagnosis of CD. Our results also demonstrated that the diagnostic model established with the selected hub genes has good diagnostic performance.

Although this study developed a new CD diagnostic model based on the GEO database, there are limitations. Our sample size was small and there was a mismatch between CD patients and controls. In the future, we will conduct external studies and validation in other independent cohorts of CD patients to evaluate generalization and robustness of this new CD diagnostic model. Secondly, we will construct an animal model and perform a large number of cellular experiments (such as the function of genes in the pathway) to further investigate the specific role of Hub genes in CD. In order to provide more in-depth and comprehensive support for future CD diagnosis research, a comprehensive analysis was conducted by combining experimental data and clinical data.

## Supplementary Materials

Supplementary Material

## Figures and Tables

**Supplementary:**
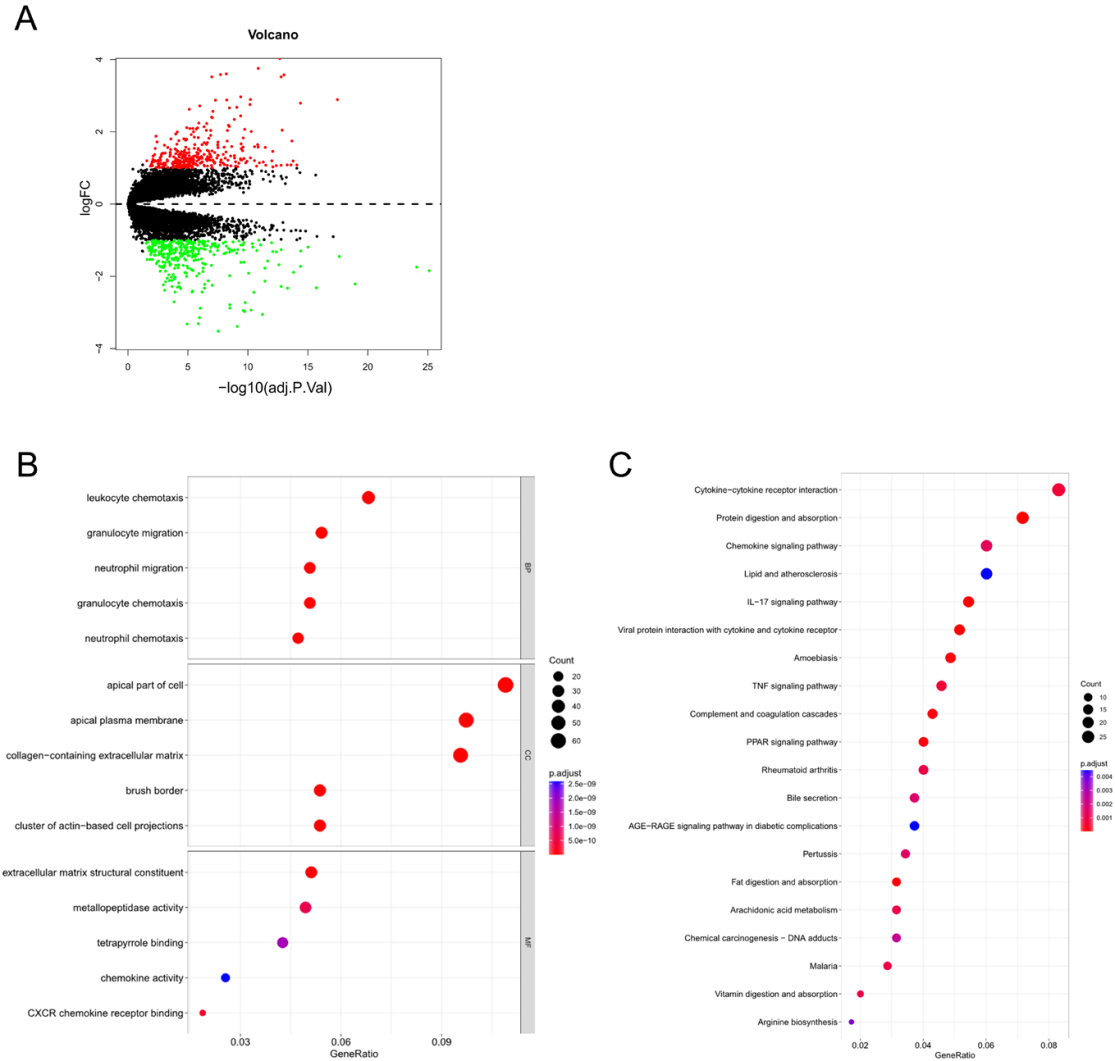
Supplementary Figure 1.

**Supplementary Table 1.** 651 DEGs between CD and control samples

https://docs.google.com/spreadsheets/d/1sGMqtCK_kHvocT5TjthalC2bMjqEp1E6pmsir6s8zaw/edit?usp=sharing

**Supplementary Table 2.** 381 ME turquoise module genes

https://docs.google.com/spreadsheets/d/1sGMqtCK_kHvocT5TjthalC2bMjqEp1E6pmsir6s8zaw/edit?usp=sharing

**Supplementary Table 3.** Coefficients of the eight module genes analyzed by LASSO regression

https://docs.google.com/spreadsheets/d/1sGMqtCK_kHvocT5TjthalC2bMjqEp1E6pmsir6s8zaw/edit?usp=sharing

## Data Availability

The data that support the findings of this study are available on request from the corresponding author.
