# Supplementary material for: Identification of Potential Functional Modules and Diagnostic Genes for Crohn’s Disease Based on Weighted Gene Co-expression Network Analysis and LASSO Algorithm
Source: Turk J Gastroenterol. 2025 Jan 6;36(4):209–18. doi: 10.5152/tjg.2025.23605 (PMC12001489; doi:10.5152/tjg.2025.23605)
Supplement: Supplementary Material [file supplementary_material.pdf]

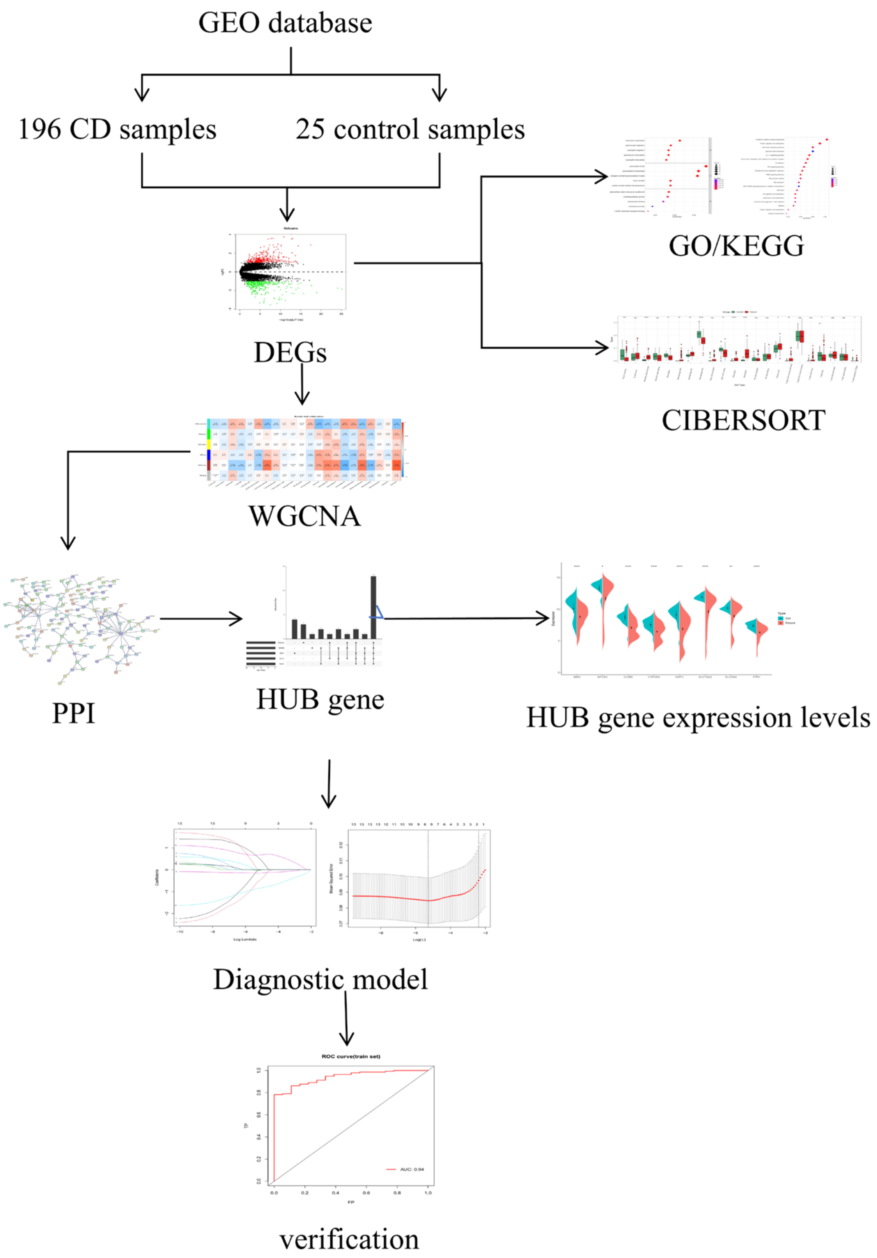

Supplementary Figure 1.

Supplementary Table 1. 651 DEGs between CD and control samples

https://docs.google.com/spreadsheets/d/1sGMqtCK_kHvocT5TjthalC2bMjqEp1E6pmsir6s8zaw/edit?usp=sharing

Supplementary Table 2. 381 ME turquoise module genes

https://docs.google.com/spreadsheets/d/1sGMqtCK_kHvocT5TjthalC2bMjqEp1E6pmsir6s8zaw/edit?usp=sharing

Supplementary Table 3. Coefficients of the eight module genes analyzed by LASSO regression

https://docs.google.com/spreadsheets/d/1sGMqtCK_kHvocT5TjthalC2bMjqEp1E6pmsir6s8zaw/edit?usp=sharing
